# Prevalence of food addiction in children and adolescents: A systematic review and meta‐analysis

**DOI:** 10.1111/obr.13183

**Published:** 2021-01-06

**Authors:** Mir Saeed Yekaninejad, Negin Badrooj, Fardis Vosoughi, Chung‐Ying Lin, Mac N. Potenza, Amir H. Pakpour

**Affiliations:** ^1^ Department of Epidemiology and Biostatistics, School of Public Health Tehran University of Medical Sciences Tehran Iran; ^2^ Department of Community Nutrition, School of Nutritional Sciences and Dietetics Tehran University of Medical Sciences (TUMS) Tehran Iran; ^3^ Department of Orthopaedic and Trauma Surgery, Shariati Hospital and School of Medicine Tehran University of Medical Sciences Tehran Iran; ^4^ Institute of Allied Health Sciences, National Cheng Kung University Hospital, College of Medicine National Cheng Kung University Tainan Taiwan; ^5^ Departments of Psychiatry and Neuroscience and the Child Study Center, School of Medicine Yale University New Haven Connecticut USA; ^6^ Connecticut Council on Problem Gambling Wethersfield Connecticut USA; ^7^ Social Determinants of Health Research Center Research Institute for Prevention of Non‐Communicable Diseases, Qazvin University of Medical Sciences Qazvin Iran; ^8^ Department of Nursing, School of Health and Welfare Jönköping University Jönköping Sweden

**Keywords:** addictive behaviors, adolescent, child, food addiction, obesity, prevalence, systematic review

## Abstract

Food addiction (FA) has been as a construct that is associated with childhood obesity. However, relatively little is known regarding the prevalence of FA among children and adolescents. An instrument designed to assess FA among youth, the Yale Food Addiction Scale for Children and Adolescents (YFAS‐C), has been developed and used to estimate FA prevalence among pediatric populations. The present systematic review and meta‐analysis aimed to synthesize the results of FA prevalence among youth. Using keywords related to FA and children to search PubMed, Embase, Scopus, and Web of Science, we identified and analyzed 22 cross‐sectional studies. No longitudinal studies were identified in the search. Meta‐analysis with Freeman‐Tukey Double Arcsine transformation was conducted to estimate FA prevalence. Meta‐regression was applied to understand whether weight status (i.e., data from community samples vs. overweight/obese samples) is associated with FA. Eligible studies (*N* = 22) were analyzed using 6,996 participants. The estimated FA prevalence was 15% (95% CI 11–19%) for all samples, 12% (95% CI 8–17%) for community samples, and 19% (95% CI 14–26%) for overweight/obese samples. Meta‐regression indicated that weight status was associated with FA severity (*p* = 0.002) and marginally with FA prevalence (*p* = 0.056). Healthcare providers should consider and address the high FA prevalence among pediatric population.

## 1 INTRODUCTION

Childhood obesity is health challenge globally with increasing prevalence^1^ and associated morbidity.^2^, ^3^ Poor health associated with obesity include physical and psychological factors among individuals^4^, ^5^, ^6^, ^7^, ^8^ and economic burdens for societies.^9^, ^10^ Therefore, finding ways to decrease the prevalence of childhood obesity is an important topic. In order to address childhood obesity, several prevention and treatment approaches have been discussed, particularly as obesity is difficult to treat perhaps given heterogeneous etiologies.^11^ One potential factor, food addiction (FA), has attracted attention.^11^, ^12^, ^13^, ^14^, ^15^

FA has been a debated concept,^16^ with some people questioning its validity.^15^ However, the development and refinement of the Yale Food Addiction Scale (YFAS)^17^, ^18^ have supported the clinical relevance of defining FA. Although FA has not been recognized or defined by the Diagnostic and Statistical Manual of Mental Disorders fifth edition (DSM‐5),^19^ the YFAS follows criteria of substance use disorders proposed by the DSM to make provisional FA diagnoses.

Specifically, the YFAS characterizes food addiction via the following items and criteria: Items 1 to 3 assess *food is taken in larger amount and for longer period than intended* (Criterion 1); Items 4, 17, 18, and 25 assess *persistent desire or repeated unsuccessful attempts to quit* (Criterion 2); items 5 to 7 assess *much time is spent to obtain and to eat food or to recover from its effects* (Criterion 3); Items 8 to 11 assess *important social, occupational, or recreational activities are given up or reduced because of food addiction* (Criterion 4); Item 21 assesses *food is continued to be used despite knowledge of adverse consequences* (Criterion 5); Items 22 and 23 assess *tolerance* (Criterion 6); Items 12 to 14 assess *characteristic withdrawal symptoms* and *food is taken to relieve withdrawal* (Criterion 7); and Items 15 and 16 assess *clinically significant impairment or distress* that is not listed in the the inclusionary criteria for substance‐use disorders but are rather mentioned prior to the specific inclusionary criteria. A 5‐point Likert scale (0 = *never*; 4 = *always*) is applied to all 18 YFAS items, and a dichotomous (*yes/no*) scale is first used for the seven items. Following this, all the items are converted dichotomously (0 = *no*; 1 = *yes*) according to specific scoring thresholds for each item. Using the converted dichotomous scores, a symptom count scoring version (ranging between 0 and 7) and a diagnostic scoring version (having 3 or more criteria met plus having clinically significant impairment or distress) can be generated.

Recent neurobiological studies provide insight into potential mechanisms underlying FA.^12^, ^14^ Given the health relevance, more research on FA is needed.^13^, ^15^ For example, literature on adults found that FA is associated with weight gain, obesity, psychological distress such as anxiety and depression, and eating disorders.^13^, ^16^, ^20^, ^21^ With respect to understanding the public health impact of FA, an assessment of its prevalence is important.

Currently, several reviews have evaluated the prevalence of FA.^20^, ^21^, ^22^, ^23^ Meule^22^ concluded that studies of FA prevalence were rare at the time of his review. He reported that FA prevalence is elevated in individuals with obesity, particularly in those with binge eating disorder. His review suggested an arguably high prevalence (~10%) among individuals regardless of their weight status. Pursey et al.^23^ later focused on FA prevalence when assessed with the YFAS. Their weighted mean prevalence of the FA according to the YFAS FA diagnosis indicates a prevalence rate (19.9%) nearly doubled what Meule^22^ concluded. Pursey et al.^23^ additionally stated that higher prevalence was found among individuals aged over 35 years, females, and those with obesity. Imperatori et al.,^20^ though not using systematic review or meta‐analysis, gathered findings from FA prevalence studies and concluded that FA is more prevalent among individuals having eating disorders (compared with those not having eating disorders) and those with obesity (compared with those of lean/normal weight). In addition, Imperatori et al.^20^ reported on limited research studying FA among children and adolescents; the prevalence was between 7.2% and 29% in these studies. More recently, Penzenstadler et al.^21^ systematically reviewed studies using the YFAS and reached a similar conclusion to other reviews^20^, ^22^, ^23^: namely, FA prevalence is relatively high. In addition, Penzenstadler et al.^21^ noted that FA was associated with higher body mass index (BMI) and having eating disorders.

Although FA studies are increasing and initial information regarding FA prevalence has been disseminated, little is known about FA prevalence among children and adolescents. Indeed, among the reviews mentioned above, only Imperatori et al.^20^ have reported information on children and adolescents. However, the information of FA prevalence in children and adolescents reported by Imperatori et al.^20^ is limited given the following two reasons. First, Imperatori et al.^20^ did not use a systematic review or meta‐analysis to synthesize the findings. Second, few studies had investigated FA among children and adolescents when Imperatori et al.^20^ conducted the review.

In order to understand FA prevalence in children and adolescents (individuals aged below 21 years), we conducted a systematic review and meta‐analysis. Specifically, the present systematic review and meta‐analysis is now feasible because (1) a children and adolescents version of the YFAS has been developed (i.e., YFAS‐C) with promising psychometric properties (the YFAS‐C was developed via [1] rewording the sentence descriptions relevant to children and adolescents' daily livings; for example, school instead of employment is mentioned in the YFAS‐C and [2] editing the sentence descriptions for a reading level equivalent to grade 2.7)^24^; and, (2) more studies of FA among children and adolescents have been conducted. Thus, the present systematic review and meta‐analysis is both feasible and timely.

## 2 METHODS

The study was registered at PROSPERO (CRD42020142198).

### 2.1 Literature search strategy

This systematic review and meta‐analysis was performed in accordance with the Preferred Reporting Items for Systematic Reviews and Meta‐Analysis (PRISMA) checklist.^25^ A search was conducted in PubMed, Embase, Scopus, and Web of Science through August 20, 2020. The search strategy included the key terms “food addiction” or “compulsive eating” and “children” or “adolescent” or “child” or “child, preschool.” After removing the duplicate studies, 402 studies remained, and the titles and abstracts of those studies were evaluated by two independent reviewers (M.S.Y. and N.B.). Three‐hundred twenty studies were removed after this stage. The full texts of the 82 remaining articles were studied by two independent reviewers (M.S.Y. and N.B.). Disputes about the possible inclusion of studies in the meta‐analysis were resolved by consulting a third reviewer (A.H.P.). Finally, 22 studies were included in the meta‐analysis.

### 2.2 Type of study

The search included observational studies including cohort, case–control, and cross‐sectional studies.

### 2.3 Type of participants

All studies that reported prevalence of FA in children as an outcome were reviewed. Peer‐reviewed publications were included if they reported the YFAS‐C score (in children aged under 21 years). We defined 21 years as the upper limit of the children's age because the statement made by the American Academy of Pediatrics identified the upper age limit of pediatrics as 21 years.^26^ Only publication in English was included. Other systematic reviews and letters to editors were excluded. Single case reports or case series and findings of clinical trials were excluded.

### 2.4 Clinical appraisal

After finalizing the included papers, each was assessed for quality by calculating the Newcastle‐Ottawa score (NOS) via the Newcastle‐Ottawa checklist. The Newcastle‐Ottawa checklist assesses quality of a study by considering eight domains. It considers the use of appropriate statistical tests, assessment of outcomes, controlling for potentially confounding factors, ascertainment of exposure, approaching non‐respondents, sample size, representativeness of the sample, and clear goals as main determinants of a study's quality. Highest quality papers get the maximum score (16), and those with a NOS of 5 or less were excluded.^27^

### 2.5 Outcome measures

The prevalence of FA among children as measured by the YFAS‐C score was calculated, along with the mean YFAS‐C score and the correlations between BMI *z*‐scores and FA.

### 2.6 Data synthesis and statistical analysis

After extensive review, a meta‐analysis was conducted using the STATA software version 15.0 (StataCorp LLC). The STATA metaprop and metan commands were used. Forest plots are provided to illustrate the point and interval estimations. In meta‐analysis for prevalence, Freeman‐Tukey Double Arcsine transformation was used to stabilize the variances. The metaninf command was used for sensitivity analysis via evaluating the effect of each study on overall estimates. The *I* ^2^ index and Egger's and Begg tests were performed to determine heterogeneity and publication bias. Funnel plots are also provided in this regard.

Studies were divided into community samples and overweight/obese samples among studies reporting this information. Stratified analysis for prevalence, mean score of YFAS‐C and linear correlation of FA and BMI *z*‐score were performed according to this categorization.

In all analyses, BMI *z*‐scores were used if available. BMI *z*‐scores were available or calculated from percentile values for 15 studies (please see Table 1 for detailed information). BMI *z*‐scores were based on objective height and weight measures in 11 studies, and in four studies, height and weight values were based on self‐reports (please see Table 1 for detailed information). The mean percentiles of BMI values of three studies (please see Table 1 for detailed information) were transformed to BMI *z*‐scores using the inverse cumulative distribution function of normal distribution.

**TABLE 1:** Information on the included 22 studies

	First author, year	Continent	Country	Target sample	Sample size	Mean age (SD)/age range	Male	YFAS‐C: Mean (SD)	FA prevalence (%)	BMI *z*‐score		Correlation coefficient of FA and BMI *z*‐score	NOS
										Mean (SD) /range	Measurement method		
1	Gearhardt, 2013^24^	America	USA	Community sample	75	8.32 (2.78) /4–16	43	2.00 (1.81)	7.20	0.53 (0.46)	Self‐report	N A	10
2	Laurent, 2016^28^	America	USA	Community sample	50	11.50 (1.12)/9–14	26	NA	4.00	0.74 (0.91)	NA	NA	9
3	Ahmed, 2016^29^	Africa	Egypt	Community sample	400	NA	196	NA	12.00	NA	NA	NA	9
4	Ahmed, 2017^30^	Africa	Egypt	Community sample	401	13.98 (1.93)/11–18	180	NA	20.20	1.48 (1.43)	Objective	NA	11
5	Ahmed, 2017^31^	Africa	Egypt	Community sample	801	14.40 (1.70) /11–18	372	NA	15.70	NA	NA	NA	12
6	Burrows, 2017^32^	Australia	Australia	Community sample	150	8.20 (2.30)/5–12	76	2.20 (2.10)	22.70	1.10 (1.90)	Self‐report	NA	10
7	Richmond, 2017^33^	America	USA	Community sample	70	8.24 (2.70) /4–16	40	NA	NA	0.57 (0.47)	Self‐report	0.290	10
8	Mies, 2017^34^	Europe	Netherlands	Community sample	339	NA	NA	1.30 (1.60)	5.90	NA	NA	NA	10
9	Tompkins, 2017^35^	America	USA	Overweight and obese sample	26	14.00 (1.90) /12–18	12	2.35 (1.80)	30.70	2.1 (0.4) /1.17–2.94	Objective	NA	8
10	Rose, 2017^36^	America	USA	Overweight and obese sample	69	16.50 (1.50)/13–21	24	2.92 (3.27)	16.20	NA	NA	NA	8
11	Naghashpour, 2018^37^	Asia	Iran	Community sample	209	9.60 (1.70)/7–13	147	NA	17.30	0.46 (1.60)	Objective	0.157	11
12	Magyar, 2018^38^	Europe	Hungary	Community sample	191	15.10 (1.70)/8–18	109	1.70 (1.20)	8.90	NA	NA	NA	8
13	Schulte, 2018^39^	America	USA	Overweight and obese sample	179	13.75 (1.35)/12–16	59	2.11 (1.75)	10.10	2.29	NA	0.190	10
14	Schiestl, 2018^40^	America	USA	Community sample	127	14.38 (1.02) /13–16	61	NA	NA	0.95 (0.89)	Objective	0.330	9
15	Borisenkov, 2018^41^	Europe	Russia	Community sample	1,144	15.20 (1.70)/12–18	531	1.5 (2.0)	4.5	−0.06	Self‐report	0.231	10
16	Filgueiras, 2019^42^	America	Brazil	Overweight and obese sample	138	9.57/9–11	64	NA	24	1.94 (0.67)	Objective	NA	11
17	Domoff, 2020^43^	America	USA	Community sample	193	14.57 (1.08)	49	NA	NA	0.79	Objective	0.320	9
18	Hardee, 2020^44^	America	USA	Community sample	76	14.30 (2.80)	44	0.7 (1.0)	NA	NA	NA	NA	9
19	Ahorsu, 2020^45^	Asia	Iran	Overweight and obese sample	1,497	15.10 (6.00)/13–18	684	2.7 (2.0)	23.9	2.2 (0.5)	Objective	0.392	11
20	Valtier, 2020^46^	America	Mexico	Community sample	245	15.83 (0.69)/15–17			19.6	NA	NA	NA	6
21	Lin, 2020^47^	Asia	Iran	Overweight and obese sample	861	15.90 (3.20)/13–18	489	2.80 (1.3)	25.90	2.40 (0.60)	Objective	0.305	11
22	Lin, 2020^48^	Asia	Iran	Overweight and obese sample	1,189	15.50 (1.90)/13–18		2.69 (2)	12.1	3.0 (1.0)	Objective	0.401	11

*Note*: The mean percentile of BMI values of Burrows, 2017,^32^ Filgueiras, 2019,^42^ and Tompkins, 2017,^35^ were transformed to BMI *z*‐scores using the inverse cumulative distribution function of normal distributions.

Abbreviations: BMI, body mass index; FA, food addiction; NA, not applicable; NOS, Newcastle‐Ottawa score; YFAS‐C, Yale Food Addiction Scale for Children.

Meta‐regression analysis on the effects of age, gender ratio, target population (i.e., overweight/obese samples vs. community samples), NOS score, sample size, and continent on prevalence of FA and YFAS‐C scores was performed in separate models for each covariate using metareg command in STATA.

## 3 RESULTS

### 3.1 Included studies

Extensive systematic review of the literature initially identified 400 studies. Among these, 320 were excluded after screening the titles and abstracts. Full texts of the remaining 80 papers were further evaluated. After removing adult studies, animal studies, other review articles, and those without reported YFAS‐C scores, 22 studies were included (Table 1). Sample size across studies was 6,996 participants. Kappa for the abstract review was 0.88 (95% CI; 0.77–0.98), and Kappa for the full text review was 0.92 (95% CI; 0.84–0.99). All 22 studies were cross‐sectional.^24^, ^28^, ^29^, ^30^, ^31^, ^32^, ^33^, ^34^, ^35^, ^36^, ^37^, ^38^, ^39^, ^40^, ^41^, ^42^, ^43^, ^44^, ^45^, ^46^, ^47^, ^48^ No longitudinal studies were identified in the search.

### 3.2 Quality assessment

**FIGURE 1:**
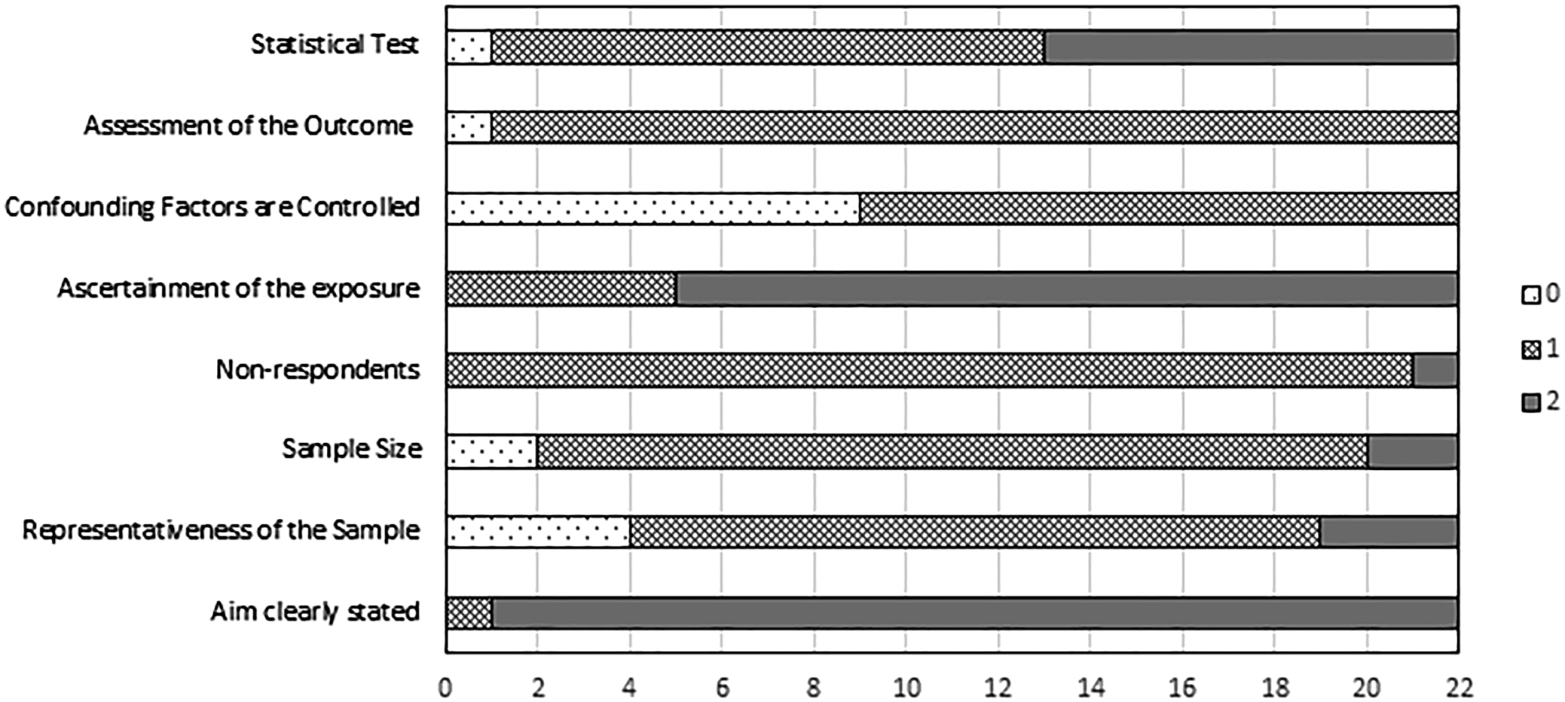
Graphical representation of the quality assessment of included studies based on Newcastle‐Ottawa score domains

The NOSs for the 22 included publications^24^, ^28^, ^29^, ^30^, ^31^, ^32^, ^33^, ^34^, ^35^, ^36^, ^37^, ^38^, ^39^, ^40^, ^41^, ^42^, ^43^, ^44^, ^45^, ^46^, ^47^, ^48^ were calculated. No article had a NOS of 5 or less (Table 1). A graphical representation of each NOS domain is provided (Figure 1).

### 3.3 Heterogeneity and publication bias

**FIGURE 2:**
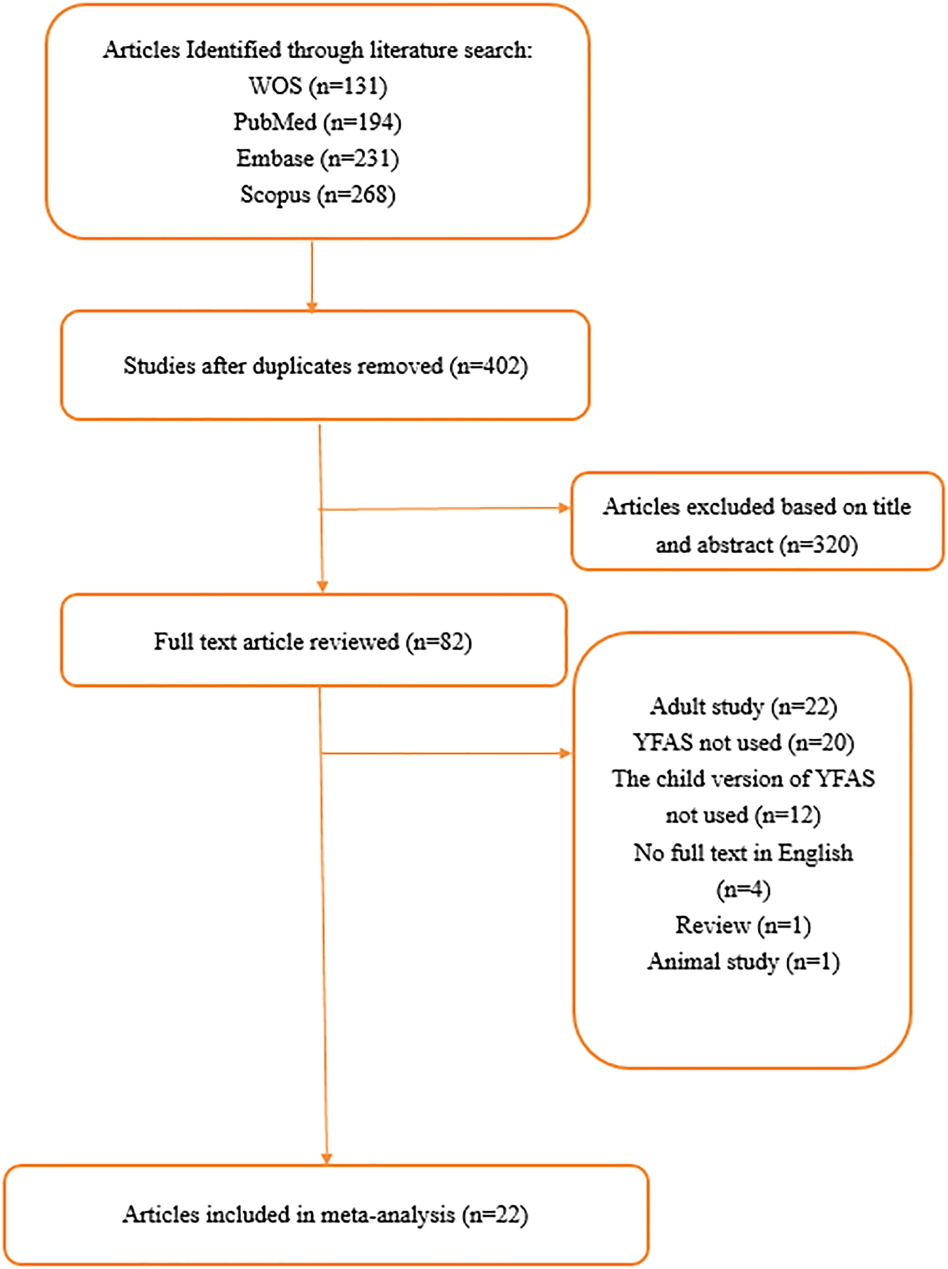
Flow chart for study selection

**FIGURE 3:**
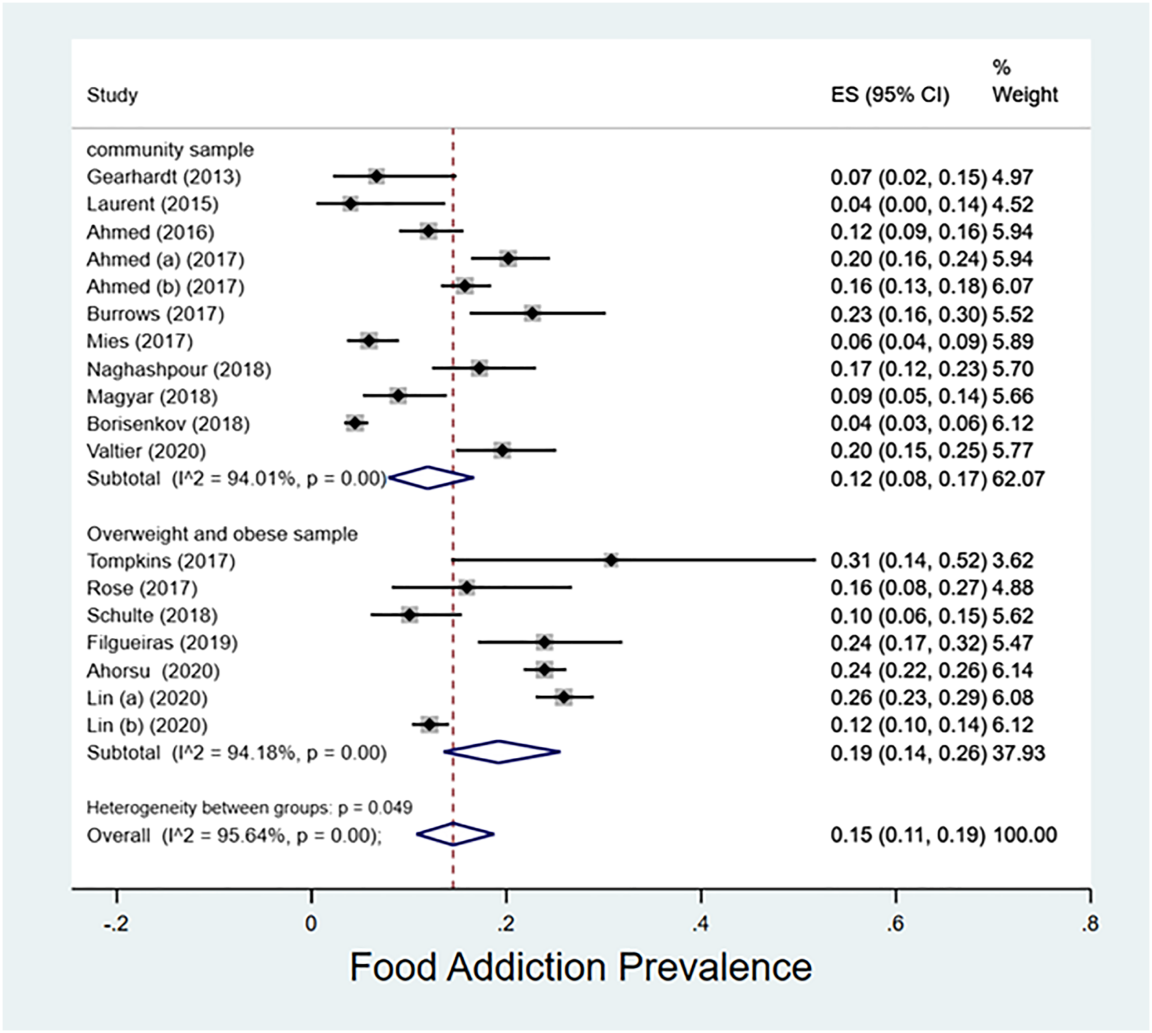
Forest plot for the prevalence of food addiction. Lin (a) (2020) is Lin CY, Cheung P, Imani V, Griffiths MD, Pakpour AH. The mediating effects of eating disorder, food addiction, and insomnia in the association between psychological distress and being overweight among Iranian adolescents. Nutrients. 2020;12(5):1371. https://doi.org/10.3390/nu12051371. Lin (b) (2020) is Lin CY, Imani V, Griffiths MD, Pakpour AH. Validity of the Yale Food Addiction Scale for Children (YFAS‐C): Classical test theory and item response theory of the Persian YFAS‐C [published online ahead of print, 2020 Jul 16]. Eat Weight Disord. 2020. https://doi.org/10.1007/s40519‐020‐00956‐x

**FIGURE 4:**
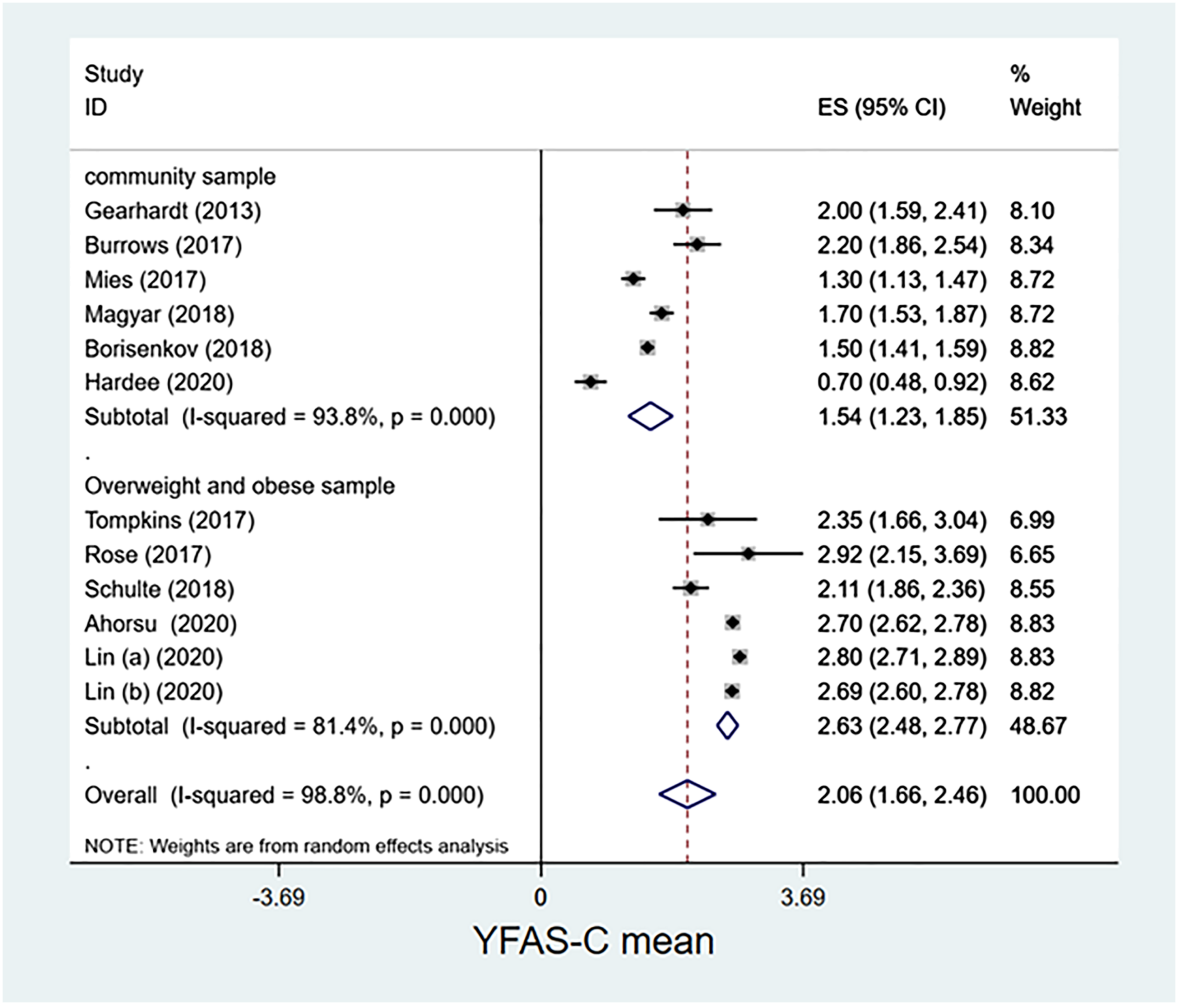
Forest plot for mean Yale Food Addiction Scale for Children scores. Lin (a) (2020) is Lin CY, Cheung P, Imani V, Griffiths MD, Pakpour AH. The mediating effects of eating disorder, food addiction, and insomnia in the association between psychological distress and being overweight among Iranian adolescents. Nutrients. 2020;12(5):1371. https://doi.org/10.3390/nu12051371. Lin (b) (2020) is Lin CY, Imani V, Griffiths MD, Pakpour AH. Validity of the Yale Food Addiction Scale for Children (YFAS‐C): Classical test theory and item response theory of the Persian YFAS‐C [published online ahead of print, 2020 Jul 16]. Eat Weight Disord. 2020. https://doi.org/10.1007/s40519‐020‐00956‐x

**FIGURE 5:**
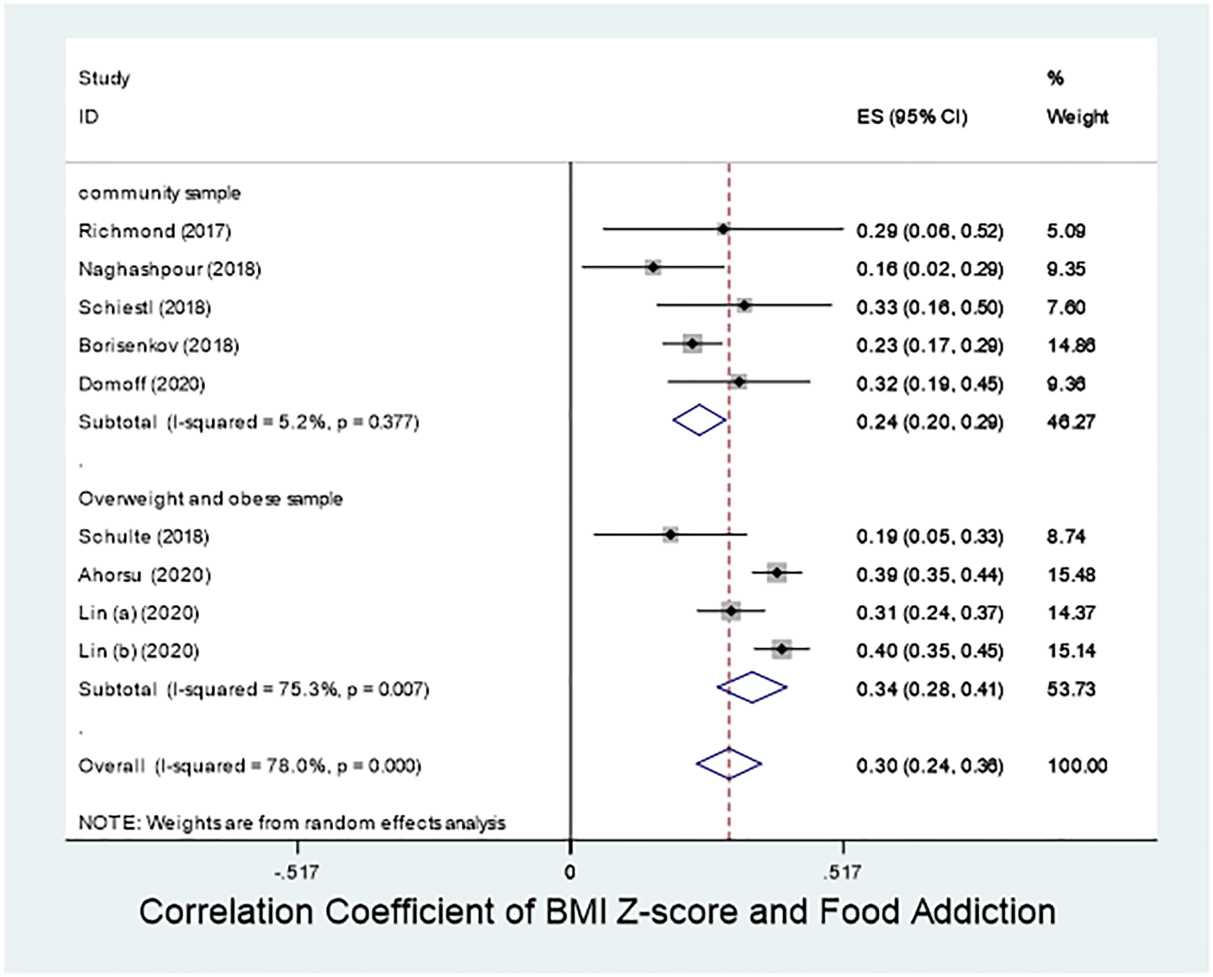
Forest plot for the correlation between BMI *z*‐score and Yale Food Addiction Score for Children (YFAS‐C). Lin (a) (2020) is Lin CY, Cheung P, Imani V, Griffiths MD, Pakpour AH. The mediating effects of eating disorder, food addiction, and insomnia in the association between psychological distress and being overweight among Iranian adolescents. Nutrients. 2020;12(5):1371. https://doi.org/10.3390/nu12051371. Lin (b) (2020) is Lin CY, Imani V, Griffiths MD, Pakpour AH. Validity of the Yale Food Addiction Scale for Children (YFAS‐C): Classical test theory and item response theory of the Persian YFAS‐C [published online ahead of print, 2020 Jul 16]. Eat Weight Disord. 2020. https://doi.org/10.1007/s40519‐020‐00956‐x

Egger and Begg tests both showed no significant publication bias for the estimated prevalence of FA (*p* = 0.084 and 0.82, respectively) (Figure 2). These tests also showed no significant publication bias for the estimated mean YFAS‐C score (*p* = 0.295 and *p* > 0.90, respectively). Heterogeneity assessed by *I* ^2^ values is depicted in each of the corresponding forest plots (Figures 3, 4, and 5).

### 3.4 Food addiction prevalence

**FIGURE 6:**
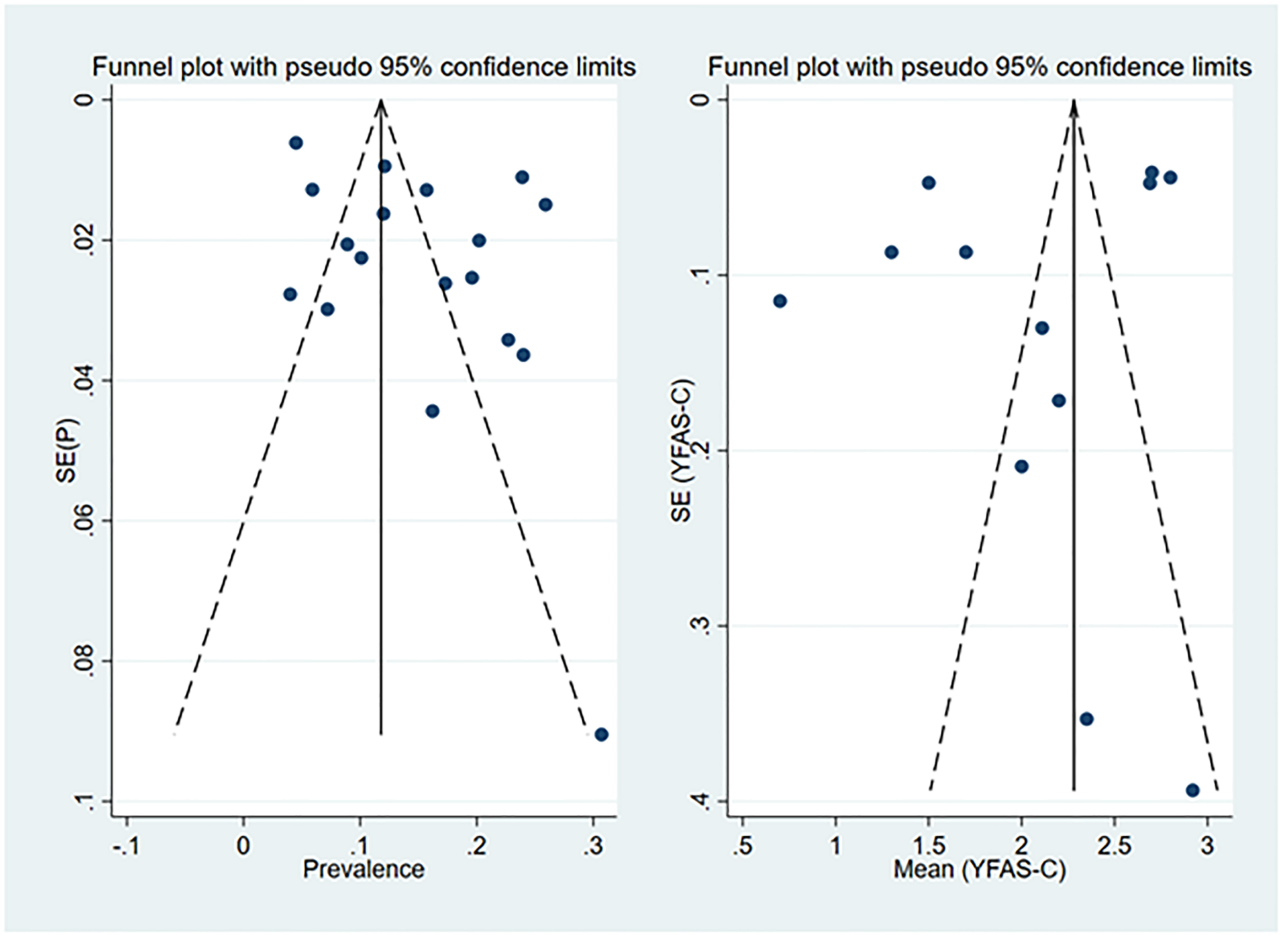
Funnel plots for the prevalence of food addiction and mean Yale Food Addiction Scale for Children scores

Prevalence of FA as determined using the YFAS‐C has been reported in 18 publications^24^, ^28^, ^29^, ^30^, ^31^, ^32^, ^34^, ^35^, ^36^, ^37^, ^38^, ^39^, ^41^, ^42^, ^45^, ^46^, ^47^, ^48^ (Table 2). The meta‐analysis estimated an overall FA prevalence at 15% (95% CI: 11–19%) among children and adolescents, with an *I* ^2^ of 95.64%. Moreover, the prevalence in the community samples was 12% (95% CI: 8–17%) and 19% (95% CI: 14–26%) in the overweight/obese samples (Figure 6).

**TABLE 2:** Prevalence of food addiction among 18 studies and sensitivity analysis

	Study omitted	Estimate	95% CI
1	Gearhardt, 2013^24^	15.51	11.47–19.56
2	Laurent, 2015^28^	15.71	11.68–19.74
3	Ahmed, 2016^29^	15.27	11.12–19.42
4	Ahmed, 2017^30^	14.74	10.71–18.77
5	Ahmed, 2017^31^	15.04	10.86–19.22
6	Burrows, 2017^32^	14.63	10.63–18.63
7	Mies, 2017^34^	15.66	11.53–19.78
8	Tompkins, 2017^35^	14.62	10.67–18.57
9	Rose, 2017^36^	15.00	10.98–19.02
10	Naghashpour, 2018^37^	14.93	10.87–18.99
11	Magyar, 2018^38^	15.45	11.35–19.54
12	Schulte, 2018^39^	15.37	11.28–19.45
13	Borisenkov, 2018^41^	15.67	12.16–19.18
14	Filgueiras, 2019^42^	14.57	10.58–18.57
15	Ahorsu, 2020^45^	14.40	10.76–18.04
16	Valtier, 2020^46^	14.79	10.75–18.82
17	Lin, 2020^47^	14.30	10.57–18.04
18	Lin, 2020^48^	15.30	10.91–19.69

### 3.5 YFAS‐C scores

The mean YFAS‐C score was calculated to be 2.06 (95% CI: 1.66–2.46) among the 12 studies^24^, ^32^, ^34^, ^35^, ^36^, ^38^, ^39^, ^41^, ^44^, ^45^, ^47^, ^48^ that reported the mean scores (Table 3). Heterogeneity (*I* ^2^) index was 98.8%. The mean YFAS‐C score in the community samples was 1.54 (95% CI: 1.23–1.85) and 2.63 (2.48–2.77) in the overweight/obese samples.

**TABLE 3:** Mean YFAS‐C score among 12 studies and sensitivity analysis

	Study omitted	Estimate	95% CI
1	Gearhardt, 2013^24^	2.07	1.65–2.48
2	Burrows, 2017^32^	2.05	1.63–2.47
3	Mies, 2017^34^	2.13	1.73–2.53
4	Tompkins, 2017^35^	2.04	1.63–2.45
5	Rose, 2017^36^	2.00	1.59–2.41
6	Magyar, 2018^38^	2.10	1.67–2.52
7	Schulte, 2018^39^	2.06	1.63–2.48
8	Borisenkov, 2018^41^	2.11	1.73–2.49
9	Hardee, 2020^44^	2.19	1.81–2.57
10	Ahorsu, 2020^45^	2.00	1.55–2.45
11	Lin, 2020^47^	1.99	1.56–2.42
12	Lin, 2020^48^	2.00	1.56–2.45

### 3.6 Correlation of food addiction with BMI *z*‐score

The correlation coefficients between FA (as determined by YFAS‐C) and BMI *z*‐scores were available for nine studies with four studies^39^, ^45^, ^47^, ^48^ having overweight and obese samples. Meta‐analysis on the correlation between BMI *z*‐scores and FA (as determined by YFAS‐C) revealed a mild to moderate correlation coefficient of 0.30 (95% CI: 0.24–0.36). Heterogeneity index (*I* ^2^) for this meta‐analysis was 78% (*p* < 0.001). Even the strongest coefficient found in the study by Lin et al.^48^ was not high (it was 0.40; 95% CI: 0.35–0.45). The correlations were 0.24 (95% CI: 0.20–0.29) in the community samples and 0.34 (95% CI: 0.28–0.41) in the overweight/obese samples.

### 3.7 Meta‐regression analyses

In the meta‐regression analysis, the target sample had a significant effect on YFAS‐C score (*p* = 0.002) and borderline significant effect on prevalence (*p* = 0.056) (Table 4). Specifically, studies on overweight/obese samples had a higher YFAS‐score and FA prevalence than did the community samples. Age (*p* = 0.637 and 0.902), BMI *z*‐score (*p* = 0.241 and 0.450), NOS score (*p* = 0.588 and 0.160), continent (*p* = 0.320 and 0.388), size of sample (*p* = 0.253 and 0.663), and sex ratio (*p* = 0.154 and 0.648) were not significantly associated with YFAS‐C score and FA prevalence.

**TABLE 4:** Meta‐regression models regarding food addiction

	Food addiction prevalence						YFAS‐C score					
	β	SE	*p*	*I* ^2^ residual (%)	Adj. *R* ^2^ (%)	tau^2^	β	SE	p	*I* ^2^ residual (%)	Adj. *R* ^2^ (%)	tau^2^
Mean BMI *z*‐score	0.033	0.403	0.450	94.6	−8.2	0.005	0.388	0.155	0.241	80.7	87.41	0.009
Mean age	0.001	0.007	0.902	96.7	−7.8	00.005	0.038	0.078	0.637	98.8	−8.80	0.463
NOS score	0.007	0.013	0.558	96.0	−2.6	0.005	0.241	0.159	0.160	97.2	17.7	0.364
Target sample	0.071	0.034	0.056	94.3	16.76	0.004	1.047	0.253	0.002	90.72	62.48	0.166
Continent	0.012	0.014	0.388	96.3	0.88	0.005	0.141	0.138	0.32	97.7	3.83	0.426
Sample size	0.001	0.000	0.663	99.5	−4.84	0.005	0.001	0.000	0.253	98.6	7.07	0.411
% of female	0.048	0.116	0.648	96.4	−5.97	0.005	1.761	1.143	0.154	98.88	10.15	0.398

*Note*: Target sample = community samples versus overweight/obese sample.

Abbreviations: Adj. *R* ^2^, adjusted *R* ^2^; BMI, body mass index; NOS, Newcastle‐Ottawa score; YFAS‐C, Yale Food Addiction Scale for Children.

### 3.8 Sensitivity analysis

Sensitivity analysis showed that no considerable impacts were found in the estimates of FA prevalence and YFAS‐C scores after omitting any of the included studies (Tables 2 and 3).

## 4 DISCUSSION

Given limited information on the prevalence of FA among children and adolescents, the present systematic review and meta‐analysis assessed FA in this population. After identifying 22 studies^24^, ^28^, ^29^, ^30^, ^31^, ^32^, ^33^, ^34^, ^35^, ^36^, ^37^, ^38^, ^39^, ^40^, ^41^, ^42^, ^43^, ^44^, ^45^, ^46^, ^47^, ^48^ with a total of 6,996 participants, the prevalence of FA found in children and adolescents was relatively high (15%; 95% CI 11–19%). Moreover, the analyzed studies were published recently, with the earliest study published in 2013^25^ and the remaining 21 studies published in the past 5 years.^28^, ^29^, ^30^, ^31^, ^32^, ^33^, ^34^, ^35^, ^36^, ^37^, ^38^, ^39^, ^40^, ^41^, ^42^, ^43^, ^44^, ^45^, ^46^, ^47^, ^48^ Therefore, the prevalence estimates reported here represent recent/current findings. Additionally, FA prevalence tended to be higher and YFAS‐C scores were higher in children and adolescents with overweight/obesity (prevalence of 19% and score of 2.63) as compared with their community‐based counterparts (prevalence of 12% and score of 1.54). Further meta‐regression analysis revealed that the difference of FA prevalence approached statistical significance (*p* = 0.056) and that of the YFAS‐C score reached statistical significance (*p* = 0.002).

The relatively high FA prevalence found in children and adolescents is comparable with that in adults.^21^, ^22^, ^23^ Therefore, FA appears relevant to both pediatric and adult populations. Indeed, Pursey et al.,^23^ Imperatori et al.,^20^ and Penzenstadler et al.^21^ all reported high FA prevalence in adult populations. Moreover, similar to adults, a trend of higher FA prevalence was found among children with overweight/obesity when compared with those without overweight/obesity in the community. Specifically, higher FA prevalence was found in adults with obesity than those without obesity in a meta‐analysis^23^ and two narrative reviews.^20^, ^21^ Our meta‐analysis on children and adolescents resonates with these findings and extends them findings to a younger age group. In addition to FA prevalence, higher YFAS‐C score found in our meta‐analysis supports the importance to considering FA in youth. Therefore, healthcare providers, policy makers, parents, and other stakeholders should attend to FA in youth.

However, unlike weight status, other factors proposed to be relevant to FA prevalence by Pursey et al.^23^ in adults were not significantly associated with FA prevalence in our pediatric population. Pursey et al.^23^ found that adults aged over 35 years and being female were linked to FA. However, our meta‐regression results showed non‐significant effects of age and gender on both FA prevalence and YFAS‐C scores. Thus, when considering FA among pediatric populations, healthcare providers and other stakeholders should notice that boys and girls may share comparable risks for developing FA. However, this is an interesting finding as ample evidence shown in the literature indicates that girls as compared with boys have higher prevalences of eating disorders.^49^, ^50^, ^51^ Given that FA has been found to be associated with eating disorders,^13^ future studies are warranted to examine the mechanisms why gender‐related differences have been found in eating disorders but not FA among pediatric population. As age did not influence results, comparable risks for FA may exist throughout much of childhood and adolescence. Thus, these findings suggest that resource allocation may be best evenly distributed across genders and child/adolescent age groups, although this possibility warrants more study.

All analyzed studies used the same instrument, the YFAS‐C, to assess FA. The YFAS‐C is a “gold standard” in assessing FA among children and adolescents. Specifically, it has been developed with rigorous methodologies, including the adoption of addiction criteria proposed in the DSM‐5^19^ and the descriptions modified for use among youth.^24^ Moreover, the strong psychometric properties of the YFAS‐C have been reported in many studies using multiple testing methods, including classical and modern test theories.^24^, ^38^, ^39^, ^40^, ^48^ Moreover, using the same instrument to assess FA across the synthesized studies included in the present meta‐analysis helps to ensure measurement quality. Therefore, together with the nonsignificant publication bias suggested by the Egger and Begg tests, we have confidence that the estimated FA prevalence and YFAS‐C score are very reliable.

The present systematic review and meta‐analysis has the following strengths. First, the comprehensive search uses multiple major databases (PubMed, Embase, Scopus, and Web of Science) and recommended keywords (identified according to the PECO framework). Therefore, the included studies are relevant to the study aims. Second, rigorous methodology was adopted in this systematic review and meta‐analysis. We used NOS to verify quality assurance; that is, only publications passing a specific study quality threshold were included. Moreover, publication bias, meta‐regression, and sensitivity testing were performed. Therefore, there is considerable confidence regarding the accuracy of the estimates in our meta‐analysis. Third, as mentioned earlier, all included studies used the same instrument with promising psychometric properties (i.e., the YFAS‐C). Therefore, the present systematic review and meta‐analysis has little instrument measurement bias.

There are some limitations in this study. First, causality between weight status and FA prevalence cannot be determined by our meta‐regression findings because all analyzed studies were cross‐sectional. Longitudinal studies are needed to understand better temporal relationships between FA and weight status. Additionally, the present systematic review and meta‐analysis included no randomized controlled trials. As such, relative impacts of targeting in treatment FA on weight status or weight status on FA cannot be evaluated and should be examined in future studies. Second, although the YFAS‐C is designed for assessing FA in youth and its consistent use across included studies is an advantage of the present systematic review and meta‐analysis, it is based on self‐report. Thus, general limitations of self‐report measures should be considered. Specifically, we cannot control biases of social desirability and inaccurate responses. Third, not every included study reported the BMI *z*‐score and provided information regarding whether the studied sample was overweight/obese or not. Therefore, we only relied on some studies providing such information to estimate effects of BMI *z*‐scores and weight status on FA prevalence and YFAS‐C scores. Lastly, although the included studies were located across the continents of America, Africa, Europe, Australia, and Asia, some specific areas (e.g., East and South Asia, North Europe) did not have information on this topic. Therefore, future studies are needed in these regions.

Together with the consideration of the aforementioned limitations, future studies could consider the following steps to provide additional understanding of FA impacts on children and adolescents. First, a recent study^52^ found that the YFAS was the strongest predictor of poor response to weightloss treatment among adults; however, it is unclear whether the same results can be replicated among children. Notably, there is a dearth of research investigating how the YFAS‐C predicts treatment outcomes in children. Second, as reported by our findings, there are no longitudinal studies on this topic currently. Therefore, long‐term studies are needed to observe whether YFAS‐C predicts weight gain over time. This information would also be beneficial for healthcare providers to design appropriate prevention programs targeting weight gain. Third, studies investigating differences in YFAS‐C prevalence across countries would be an important future direction as well.

## 5 CONCLUSION

In conclusion, FA is an important topic among youth. With the relatively high prevalence of FA among children and adolescents found in the present systematic review and meta‐analysis, healthcare providers, policymakers, and other stakeholders should design appropriate interventions to address FA in this age group. Moreover, higher estimates of FA were observed among children and adolescents with overweight/obesity as compared with lean/normal‐weight individuals. Thus, targeted interventions may be particularly relevant to children and adolescents with overweight/obesity.

## CONFLICT OF INTEREST

We declare no conflicts of interest. Dr. Potenza has the following disclosures. He has consulted for and advised Game Day Data, the Addiction Policy Forum, AXA, Idorsia, and Opiant/Lakelight Therapeutics; received research support from the Veteran's Administration, Mohegan Sun Casino, and the National Center for Responsible Gaming (now the International Center for Responsible Gambling); participated in surveys, mailings, or telephone consultations related to addictions, impulse‐control disorders or other health topics; consulted for law offices and gambling entities on issues related to impulse control and addictive disorders; provided clinical care in the Connecticut Department of Mental Health and Addiction Services Problem Gambling Services Program; performed grant reviews for the National Institutes of Health and other agencies; edited journals and journal sections; given academic lectures in grand rounds, CME events and other clinical/scientific venues; and generated books or chapters for publishers of mental health texts. Other authors declare that they have no disclosures.
